# Psychometric properties of the Chinese version of the Gudjonsson compliance scale: scale validation and associations with mental health

**DOI:** 10.1186/s12889-024-17970-8

**Published:** 2024-02-14

**Authors:** Yaming Hang, Gisli H. Gudjonsson, Yingying Yao, Yi Feng, Zhihong Qiao

**Affiliations:** 1 Beijing Key Laboratory of Applied Experimental Psychology, National Demonstration Center for Experimental Psychology Education, Faculty of Psychology, Beijing Normal University, No19 Xiniekouwai Street, Haidian District, Beijing, 100875 China; 2 Department of Psychology, Institute of Psychiatry, Psychology & Neuroscience, London, England; 3 Counselling and Education Centre, Student Affairs Department, Xiamen University, Fujian, China; 4 Mental Health Center, Central University of Finance and Economics, No.39 South College Road, Haidian District, Beijing, 100081 China

**Keywords:** Compliance, Scale validation, Psychometric properties, Mental health, China

## Abstract

**Background:**

Trait compliance involves people reacting favorably to demands made by others across different situations. This may lead to susceptibility to external pressures, exploitation, and manipulation. Moreover, trait compliance was found to correlate with various mental health outcomes, such as depression and anxiety. The Gudjonsson Compliance Scale (GCS) is an efficient tool for assessing trait compliance in Western contexts. To date, no study has validated the psychometric properties of the GCS in Chinese populations.

**Methods:**

Two college student samples from China were recruited. The first sample (*N* = 4,276) was used to conduct exploratory factor analysis. The second (*N* = 4,356) was used to perform a confirmatory factor analysis. The reliability, measurement invariance, and correlational tests were conducted on the two combined samples.

**Results:**

The Chinese GCS showed a 3-factor structure, with two items deleted. Reliability was supported by moderate-to-good internal consistency of the three-factor scales and good internal consistency on the full scale. Strong measurement invariance across sex, ethnicity, and group recruitment was supported. Scores of the total scale and factor scales were found to significantly associated with several mental health problems.

**Conclusions:**

The Chinese version of the GCS appears to be a valid and reliable instrument for measuring trait compliance and could promote both the assessment and research on compliance in Chinese population.

## Background

Compliance is a core concept in social influence studies. It involves people reacting favorably to demands or requests made by others [1]. It refers to the tendency of an individual to go along with propositions, requests, or instructions for some immediate instrumental gain [2]. Compliance can be conceptualized in two different ways: situational and trait. Situational compliance is regarded as a behavioral response to a particular situation, whereas trait compliance is viewed as a personality trait [3, 4]. Trait compliance is related to an individual’s susceptibility to give in to pressure in various situations, and is positively correlated with situational compliance in both personal and impersonal relationships [5]. Situational compliance has been widely studied [6, 7], whereas trait compliance is a new perspective in relatively recent studies on compliance [8]. Investigating the nature of trait compliance is important, as high trait compliance can render individuals susceptible to external pressures, exploitation, and manipulation [9], and in certain cases, lead to false confessions [10, 11].

To assess individuals’ trait compliance, the 20-item Gudjonsson Compliance Scale (GCS) was developed [8]. Three distinct underlying dimensions emerged in the original factor analysis of the GCS, including difficulty with pressure and conflict (factor 1, comprising ten items), eagerness to please (factor 2, comprising five items), and a rather obscure third factor (factor 3) consisting of five items, which included three items on which the answer ‘false’ indicates the compliant response. Among them, factors 1 and 2 are the two major components that load most highly on the GCS [8], which overlaps with Milgram’s (1974) construct of “obedience to authority” [12]. The GCS has shown good construct and concurrent validity [8], as well as good internal consistency and test-retest reliability [13, 14], and can discriminate significantly between individuals with high and low compliance [15]. In a recent study, the GCS was found to measure different aspects of compliance across males and females [13]. To date, no study has validated the reliability and validity of the GCS in a non-Western context.

The meaning of the compliance construct and its subdimensions may vary across different cultures. This is because culture is believed to influence the pace, timing, and processes by which compliance develops [16]. In Western cultures, there is often an emphasis on independence and self-assertion. While child compliance is typically encouraged during early childhood [17], parents are advised to be sensitive to their children’s needs and to view their abilities and behaviors from a “child-centered” perspective [18]. In contrast, Chinese culture places a higher value on compliance in a more consistent and absolute manner [19]. The most common term to praise children in Chinese culture are “ting hua”, which means to obedient or listening to adults’ words. Compliance with authority is emphasized from a very young age [20], and Chinese children are encouraged to behave cooperatively and compliantly (e.g., to meet others’ expectations) in social contexts while restraining their personal desires and impulsive acts [21]. However, the underlying dimensional structure of the GCS in the Chinese culture has not been investigated. Thus, this study aimed to validate the Chinese (Mandarin) version of the GCS and explore its underlying factor structure. This effort could make compliance measurement more accessible for Chinese practitioners and facilitate the identification of mental health problems more effectively.

### The relationships between compliance and mental health

Although the GCS was originally constructed to complement the theoretical and empirical work conducted on interrogative suggestibility [8], GCS scores were also found to positively correlate with several mental health outcomes, including anxiety, depression, negative emotions, dysthymia, delusional disorder, attention deficit hyperactivity disorder, and personality disorders [3, 22, 23]. In addition, several studies have focused on the relationship between trait compliance and personality traits that are closely related to mental health, such as self-esteem, neuroticism, extraversion, and psychoticism [24, 25]. Trait compliance is significantly correlated with the susceptibility of the individual to give in to pressure in a variety of specific situations. According to Eysenck’s theory of personality [26], the unstable (anxious) introvert seems most vulnerable to giving in to pressure. This was supported by Gudjonsson et al. (2004)’s study, which found that compliance was positively correlated with neuroticism and negatively correlated with extraversion among prison inmates, college students, and university students. Consistent with the original scale development article [8], the relationships between trait compliance and these conceptually related variables (neuroticism and extraversion) were examined in order to test the convergent validity of the GCS. In addition, compliant behaviors were thought to include a component representing eagerness to please and the need to protect one’s self-esteem when in the company of others [8], and low esteem has been found to be positively correlated with and predictive of compliance [3, 25]. Thus, the relationship between trait compliance and self-esteem was examined. Similarly, as the scores of the English version of the GCS were found to correlate with mental health problems, the present study also aimed to examine the relationship between trait compliance and several mental health outcomes (anxiety, depression, negative affect, and psychological distress) as an external validation among the Chinese population.

## Methods

### Data collection

In 2020, two large samples of Chinese youth (including preppies, undergraduates, and graduates) were recruited using cluster sampling. The first sample (Wave 1) was collected in a Beijing comprehensive university, which is located in northern China. The second sample (Wave 2) was collected in a Fujian comprehensive university, which is located in southern China. All students from randomly chosen classes of the two colleges were invited to complete an online survey using a questionnaire QR code distributed by their head teachers who were in charge of their classes. Almost all the students in the chosen universities participated in our study, and the participants were from around the country. All participants remained anonymous and were informed that they could withdraw from the survey at any time during the survey. The participants provided informed consent before completing the survey. A total of 4,768 participants completed the survey in Sample 1 and 5,086 participants completed the survey in Sample 2. To ensure data quality, we applied the following exclusion criteria: (a) participants whose answers were incorrect to the attention check question, (b) participants who spent less than 1 s on each item on average, and (c) non-Chinese participants. Thus, the final samples were 4,276 for Sample 1 (mean age = 20.49 years, *SD* = 4.01, 66.5% female) and 4,356 (mean age = 20.69 years, *SD* = 3.11, 52.7% female) for Sample 2. The response rate was 87.6%.

### Translation of the scale

The adaptation of the Chinese version of the GCS was authorized by the author of the original English version. The process of translation followed the recommended procedures for cross-cultural scale adaptation. The researchers conducted initial translation by two bilingual native Chinese translators, synthesis of translation by a third bilingual Chinese translator, back translation by two bilingual native English speakers and then an expert review by several psychologists, psychiatrists and medical staff.

### Measures

#### Social-demographic variables

Demographic information including gender, age, ethnicity, education level, place of residence (i.e., city, town, and country), mental disorder history, family type, siblings and socioeconomic status were collected.

### Gudjonsson compliance scale

Trait compliance was measured by the 20-item Gudjonsson Compliance Scale [8]. The 20 GCS items were originally rotated using a default Varimax procedure and three factors were extracted: Factor 1 comprising 10 items, reflecting difficulties in coping with pressure; factor 2 comprising five items, reflecting eagerness to please and to do what is expected; and factor 3 comprising five items, with modest loadings, reflecting an obscure factor difficult to define in terms of a specific latent construct. The researchers conducted initial translation by two English-Chinese bilingual native Chinese translators, back translation by two bilingual native English speakers and then an expert review by three psychology researchers. To increase the sensitivity of the scale, the rating scale was changed from a two-point (yes/no) scale to a five-point scale ranging from 1 (totally disagree) to 5 (totally agree). Example items included “I give in easily to people when I am pressured” and “When I am uncertain about things I tend to accept what people tell me”.

### Generalized anxiety disorder-7 (GAD-7)

Anxiety was assessed by the GAD-7 [27], a self-report screening scale that has been validated in China [28]. Seven items examined how often participants had been bothered by anxiety symptoms in the past 2 weeks on a 4-point scale ranging from 0 (not at all) to 3 (nearly every day). Higher scores reflected higher anxiety symptoms. In the current study, reliability for GAD-7 was α = 0.90, indicating good internal consistency.

### Patient health questionnaire-9 (PHQ-9)

Depression was measured via the PHQ-9 [29]. This self-report screening scale has been validated in Chinese people [30]. It consists of 9 items on a scale of 0–3 (0 = not at all; 1 = several days; 2 = more than a week; 3 = nearly every day). Participants rated the frequency with which they had been bothered by depressive symptoms in the past 2 weeks for each item. Higher total scores reflected higher depressive symptoms (α = 0.85).

### Positive and negative affect schedule (PANAS)

Positive and negative affect was measured by the PANAS [31], which consists of two 10-item mood scales. Participants were asked to rate the extent to which they had experienced each particular emotion over the previous two weeks on a 5-point scale, from 1 (very slightly to not at all) to 5 (extremely). In this study, reliability (internal consistency) was α = 0.90 for the positive affect subscale and α = 0.92 for the negative affect subscale.

### Symptom checklist 90 (SCL-90)

Psychological distress was assessed by the SCL-90 [32]. It is a self-report measure consisting of 90 items that represent nine factors and seven additional questions that are configured items, primarily concerning disturbances in appetite and sleep patterns. Items are rated on a five-point scale indicating distress, ranging from 0 (not at all) to 4 (extremely). The scores for the 90 items can be added to calculate the total score. The total scores range from 0 to 360 and serve as an indicator of general psychological distress. In this study, the time of reference for the symptoms was the previous week. In the current study, reliability (internal consistency) for the SCL-90 was α = 0.98.

### Rosenberg self-esteem scale (RSES)

Self-esteem was measured by the Rosenberg Self-Esteem Scale, which consists of ten items answered on a 4-point scale from “strongly agree” to “strongly disagree” [33]. In this study, internal consistency for the RSES was α = 0.88.

### Big Five traits

Big Five personality traits (i.e., extraversion, agreeableness, openness to experiences, conscientiousness, and neuroticism) were measured with a short 10-item scale [34]. Participants were provided with 10 sets of adjectives that described personalities, such as “extraverted, enthusiastic” (i.e., extraversion) or “sympathetic, warm” (i.e., agreeableness). Participants were then asked to rate the extent to which they agreed with each characteristic on a scale ranging from 1 (strongly disagree) to 7 (strongly agree).

### Situational compliance scale (SCS)

The SCS is a 15-item inventory developed to examine situational compliance [5]. It asks about a range of situations of potential influence. The items included two different types: personal and impersonal. Personal items described situations where there is likely to be an emotional relationship between the person making and the person receiving the request (e.g., a friend or a parent), while impersonal items were related to a range of less personal situations (e.g., an intrusive salesman tries to sell the participant something he or she does not want to buy). Participants were provided with 5 five personal items and 10 impersonal items, and were asked to rate the extent to which they would comply with the request on a scale ranging from 1 (not at all) to 7 (very much). In this study, internal consistency for the SCS was α = 0.83.

### Analytic approach

To investigate the factor structure of the Chinese version of the GCS, we employed a two-step factor analysis approach. Data from Sample 1 were used to perform exploratory factor analysis (EFA) and data from Sample 2 were used to conduct confirmatory factor analysis (CFA). Reliability and other validity analyses, including measurement invariance and correlational analyses, were conducted using the two combined samples. All analyses were performed using SPSS version 23.0 and Mplus version 8.3.

First, the dimensional structure of the Chinese GCS was explored using EFA. An EFA with principal axis factoring and direct oblimin rotation was performed. The factors suggested by EFA were then examined in Sample 2 using CFA. Maximum likelihood estimation was used. To assess the global goodness of fit, the *χ*^*2*^ test of the exact model fit, comparative fit index (CFI), Tucker–Lewis index (TLI), root mean square error of approximation (RMSEA) with corresponding 90% confidence intervals, and standardized root mean square residual (SRMR) were used. Model fit was considered to be acceptable when RMSEA and SRMR were < 0.08 and CFI and TLI were > 0.90 [35]. In addition, the Akaike information criterion (AIC) and Bayesian information criterion (BIC) were calculated to assess whether one model fits better than another, with smaller values being preferred.

Second, the internal consistency of the total scale and factors was assessed using Cronbach’s alpha (α), with values greater than 0.70 indicating adequate reliability [36].

Third, measurement invariances across gender, ethnicity, and data waves were tested in sequence at the configural, metric, and scalar levels [37]. Configural invariance examines whether the overall latent factor structure is the same for both groups. Additionally, metric invariance requires item loadings to be equal across groups. Finally, scalar invariance requires the item intercepts to be the same across groups. The statistics described above were used to evaluate the goodness of fit of the invariance models. As *χ*^*2*^ is sensitive to the sample size, CFI and RMSEA were also examined. To reach a level of measurement invariance, the deterioration of CFI should not exceed 0.010 [38], and the change in RMSEA should be less than 0.015 [39].

Finally, the Pearson correlation coefficients between the Chinese GCS scores and scores of the Big Five traits, situational compliance and other variables related to mental health problems (i.e., anxiety, depression, positive and negative affect, self-esteem, and psychological distress) were calculated. Correlation coefficients of ≤ 0.30 were considered to be weak, those between 0.30 and 0.50 were moderate, and those ≥ 0.50 were deemed strong [40]. In addition, stepwise multiple regression analysis was performed to assess whether the mental health/self-esteem variables could significantly improve the prediction of compliance over the Big Five personality traits. Considering the possible bidirectional relationship between compliance and mental health outcomes, the reversed association was also examined through regression analyses to assess whether compliance could significantly improve the prediction of mental health variables over the personality variables.

## Results

### Sociodemographic characteristics

A total of 8,632 participants were included in the final two samples, with ages ranging from 14 to 48 years in Sample 1 (*M* = 20.79, *SD* = 4.01) and 15 to 43 years in Sample 2 (*M* = 20.69, *SD* = 3.11). The majority of the participants were of Han ethnicity (88.0% in Sample 1 and 91.4% in Sample 2). The sociodemographic characteristics of the two samples are presented in Table 1.

**Table 1:** Sociodemographic characteristics of the two samples

Variables	Sample 1 (*N*_*1*_ = 4276)	Sample 2 (*N*_*2*_ = 4356)	*χ*^*2*^-test/t-test	*p* value
Age (*mean* ± *SD*)	20.79 ± 4.01	20.69 ± 3.11	*t* = 1.223	0.221
Gender (*n*, (%))			Fisher’s *t* = 172.610	0.000
Male	1429 (33.4%)	2054 (47.2%)		
Female	2845 (66.5%)	2296 (52.7%)		
Others	2 (0.0%)	6 (0.1%)		
Ethnicity			*χ*^2^ = 25.776	0.000
Han	3765 (88.0%)	3980 (91.4%)		
Others	511 (12.0%)	376 (8.6%)		
Education level			*χ*^2^ = 282.149	0.000
Preppy	43 (1.0%)	188 (4.3%)		
Undergraduate	2376 (55.6%)	2060 (47.3%)		
Graduate	1764 (41.3%)	1734 (39.8%)		
Doctoral	93 (2.2%)	374 (8.6%)		
Residence			*χ*^2^ = 213.160	0.000
City	1734 (40.6%)	1228 (28.2%)		
Town	2108 (49.3%)	2309 (53.0%)		
Country	434 (10.1%)	819 (18.8%)		
Family category			*χ*^2^ = 16.998	0.000
Core family	3355 (78.5%)	3254 (74.7%)		
Others	921(21.5%)	1102 (25.3%)		
Siblings			*χ*^2^ = 150.935	0.000
None	2601 (60.8%)	2101 (48.2%)		
One	1297 (30.3%)	1631 (37.4%)		
More than one	378 (8.8%)	624 (14.3%)		
Mental disorder history			*χ*^2^ = 26.847	0.000
Yes	76 (1.8%)	156 (3.6%)		
No	4200 (98.2%)	4200 (96.4%)		
Economic status	2.96 ± 0.52	3.06 ± 0.56	*t* = -7.976	0.000
Social class status	4.78 ± 1.57	4.61 ± 1.77	*t* = 4.625	0.000
Compliance	49.90 ± 10.81	50.76 ± 10.48	*t* = -3.765	0.000

*Note.* The sample 1 was used for EFA, and the sample 2 was used for CFA. Chi-square test and t-test was applied to compare the differences of the variables between two samples. Economic status was assessed on a 5-piont scale (1 = very rich, 5 = very poor); Social class on an 11-point scale (0 = the lowest, 10 = the highest)

### Construct validity (EFA and CFA)

Data from Sample 1 were used to conduct the EFA. The results of the Kaiser Meyer-Olkin (KMO) Measure of Sampling Adequacy test and Bartlett’s test of sphericity showed that the data were suitable for EFA (KMO = 0.91; *χ*^2^ = 25726.05, *df* = 190, *p* < .001). Three factors were extracted using principal axis factoring based on the criterion of eigenvalues greater than 1, accounting for 38.2% of the total variance [41]. Items with factor loadings < 0.30 or communalities < 0.20 were considered to have a poor fit to the factor structure [42]. Item 20 (“*When I was a child I sometimes took the blame for things I had not done*”) had a factor loading of 0.14 and a communality of 0.04. Considering that its meaning in Chinese culture may differ from that of the concept of compliance, this item was removed. A second EFA was conducted with the remaining 19 items, and the results revealed that item 11 (“*Disagreeing with people often takes more time than it is worth*”) had a factor loading of 0.30 and a communality of 0.15. This item was also removed, as its meaning was far from that of compliance in Chinese culture. After the removal of the two items, the EFA results found a 3-factor structure with all 18 items having factor loadings > 0.30 and communalities > 0.20, accounting for 41.4% of the total variance. Table 2 lists the factor loadings for each item. Based on the factor labels from the English version [13] and the current item placement, three factors were identified: difficulty in coping with authority and conflict, eagerness to meet expectations, and social acceptance.

**Table 2:** Exploratory factor analysis of the Chinese version of Gudjonsson Compliance Scale (*N* = 4,276)

Items	Factor 1	Factor 2	Factor 3		Communality		
5. I tend to become easily alarmed and frightened when I am in the company of people in authority	0.73				0.46		
3. People in authority make me feel uncomfortable and uneasy	0.69				0.35		
2. I find it very difficult to tell people when I disagree with them	0.60				0.41		
6. I try very hard not to offend people in authority	0.59				0.49		
9. I believe in avoiding rather than facing demanding and frightening situations	0.57				0.46		
4. I tend to give in to people who insist that they are right	0.56				0.42		
1. I give in easily to people when I am pressured	0.55				0.40		
8. I tend to go along with what people tell me even when I know that they are wrong	0.48				0.31		
7. I would describe myself as a very obedient person	0.45				0.46		
10. I try to please others	0.35				0.45		
14. I generally try to avoid confrontation with people		0.63			0.40		
16. I try hard to do what is expected of me		0.62			0.35		
15. As a child I always did what my parents told me		0.59			0.35		
13. When I am uncertain about things I tend to accept what people tell me		0.51			0.34		
12. I generally believe in doing as I am told		0.39			0.43		
19*. I would never go along with what people tell me in order to please them			0.80		0.62		
18*. I strongly resist being pressured to do things I don’t want to do			0.73		0.48		
17*. I am not too concerned about what people think of me			0.43		0.25		

*Note.* Factor 1: Difficulty in coping with authority and conflict; Factor 2: Eagerness to meet expectations; Factor 3: Social acceptance. The second to fourth columns were factor loadings for each item

* factors that were reverse-coded

**Fig. 1:**
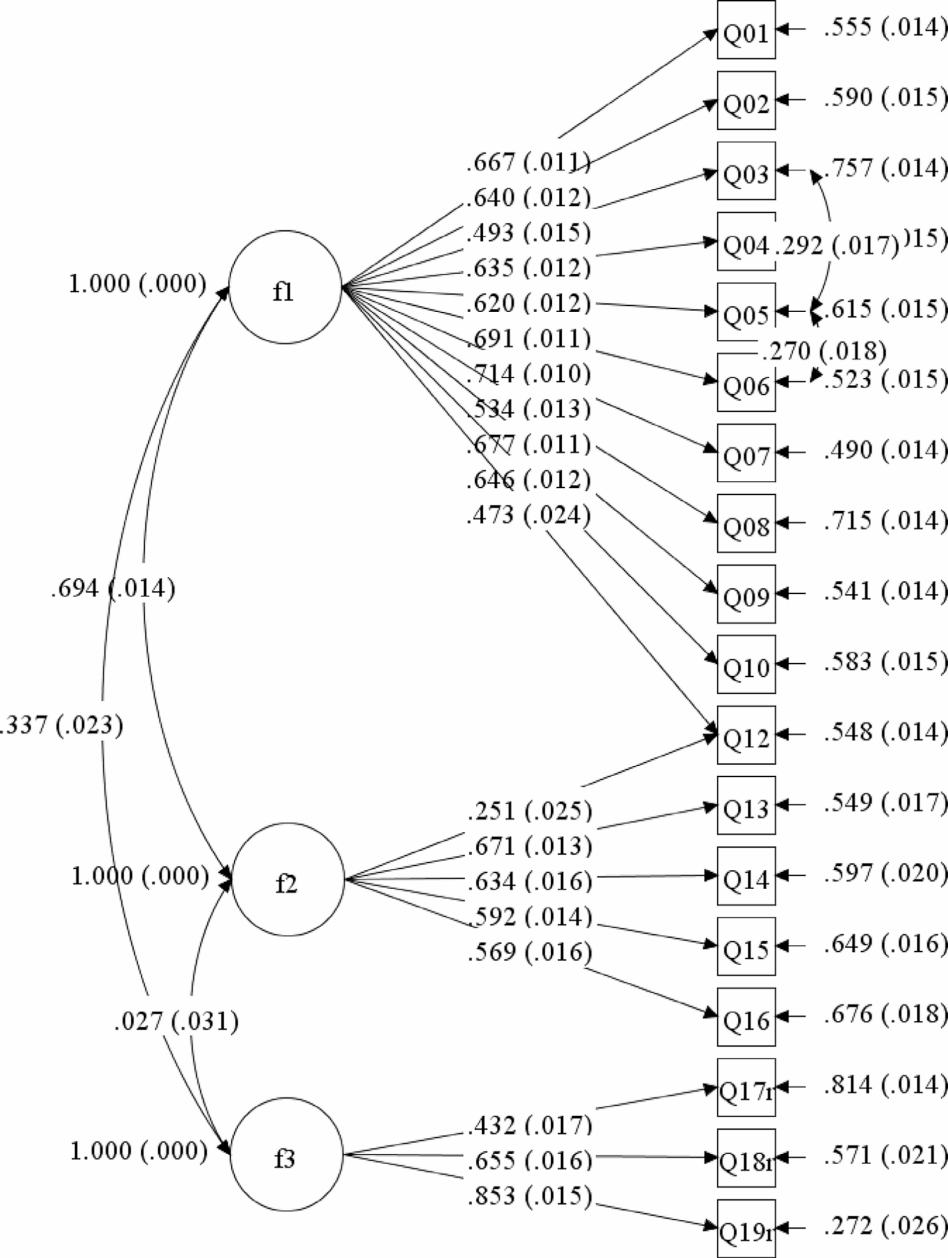
The final three-factor structure of the Chinese version of the GCS. The coefficients in the figure are standardized coefficients, with standard errors in the brackets. F1: Difficulty in coping with authority and conflict; F2: Eagerness to meet expectations; F3: Social acceptance

The 3-factor model suggested by the EFA was then tested via CFA using the data from Sample 2. The results showed that the initial model (Model 1) did not fit the data ideally (*χ*^*2*^/*df* = 25.487, RMSEA = 0.075, CFI = 0.878, TLI = 0.859, SRMR = 0.058, AIC = 202423.536, BIC = 202787.156). Based on the modification indices, we found that some items expressed comparable meanings in Chinese. Item 3 (“*People in authority make me feel uncomfortable and uneasy*”), item 5 (“*I tend to become easily alarmed and frightened when I am in the company of people in authority*”) and item 6 (“*I try very hard not to offend people in authority*”) shared similar meanings in Chinese language. Thus, we set free the covariance between these items. In addition, item 12 (“*I generally believe in doing as I am told*”) was found to have cross loadings on both the first and second factors in previous studies [8, 13], and it is concerned with both difficulty in coping with conflict and eagerness to meet expectations. Thus, item 12 was allowed to cross-load on both factors 1 and 2. As shown in Fig. 1, the final model demonstrated an acceptable fit according to the goodness-of-fit indices (*χ*^*2*^/*df* = 18.238, RMSEA = 0.063, CFI = 0.916, TLI = 0.901, SRMR = 0.049, AIC = 201417.780, BIC = 201794.159).

### Reliability

Cronbach’s alpha was calculated for the two combined samples to assess the internal consistency of the items. The alpha coefficients for each factor, the range of inter-item correlations, and the mean of each inter-item correlation are shown in Table 3. The three factors revealed moderate-to-good internal consistency (α = 0.67 ∼ 0.87), and the full scale showed good internal consistency (α = 0.88).

**Table 3:** Internal Consistency Reliability Analyses for GCS total and factor scales (*N* = 8,632)

	No. of items	Cronbach’s alpha	Range of inter-item correlations	Mean inter-item correlation
Factor 1	10	0.872	0.26~0.57	0.41
Factor 2	5	0.739	0.30~0.46	0.36
Factor 3	3	0.671	0.31~0.56	0.41
Total scale	18	0.877	-0.08~0.57	0.28

*Note.* GCS = Gudjonsson Compliance Scale. Factor 1: Difficulty in coping with authority and conflict; Factor 2: Eagerness to meet expectations; Factor 3: Social acceptance

### Measurement invariance

The configural, metric, and scalar invariance tests were each examined in sequence for the GCS in the two combined samples (see Table 4). For the three types of invariance testing, the models fit the data well for the GCS (△CFI and △RMSEA < 0.01). Overall, the results indicated that measurement invariance of the GCS across gender, ethnicity and data waves (Wave 1, *N* = 4276; Wave 2, *N* = 4356) was supported.

**Table 4:** Model fit information for invariance testing (*N* = 8,632)

Models	*χ* ^2^	*df*	*p* value	RMSEA [95% CI]	CFI	ΔRMSEA	ΔCFI
**Sex**							
Configural	6098.637	332	0.000	0.063[0.062, 0.065]	0.888		
Metric	6160.992	348	0.000	0.062[0.061, 0.064]	0.887	0.001	0.001
Scalar	6469.194	363	0.000	0.062[0.061, 0.064]	0.881	0.000	0.006
**Ethnicity**							
Configural	6616.636	332	0.000	0.066[0.065, 0.068]	0.879		
Metric	6631.575	348	0.000	0.065[0.063, 0.066]	0.879	0.001	0.000
Scalar	6652.237	363	0.000	0.063[0.062, 0.065]	0.879	0.002	0.000
**Data waves**							
Configural	6037.966	332	0.000	0.063[0.062, 0.065]	0.889		
Metric	6079.957	348	0.000	0.062[0.060, 0.063]	0.889	0.001	0.000
Scalar	6304.018	363	0.000	0.062[0.060, 0.063]	0.885	0.000	0.004

*Note.* Measurement invariance levels were assumed to be reached if (a) ΔCFI ≤ 0.10, and (b) ΔRMSEA < 0.015.

### Convergent validity and correlations between GCS and mental health

Correlations between the GCS total and factor scores, and external measures were examined in the combined sample (see Table 5). Consistent with the original version [8, 24], the GCS total and factor scores were found to be positively correlated with neuroticism, and negatively correlated with extraversion, which supported the convergent validity of the Chinese GCS. Additionally, since trait compliance was positively correlated with situational compliance across a range of situations [5], to further test the convergent validity of the GCS scores, a third sample in 2021 was recruited merely to examine the relationship between trait compliance and situational compliance as an additional external validation of the Chinese GCS. This sample consisted of 3,945 college students (mean age = 20.38 years, *SD* = 4.23, 64.3% female) with convenience sampling. The situational compliance was only measured in this sample. The correlation results showed that both the total score (*r* = .35, *p* < .001) and factor scores (factor 1: *r* = .33, *p* < .001; factor 2: *r* = .24, *p* < .001; factor 3: *r* = .24, *p* < .001) of the GCS were significantly positively correlated with situational compliance.

**Fig. 2:**
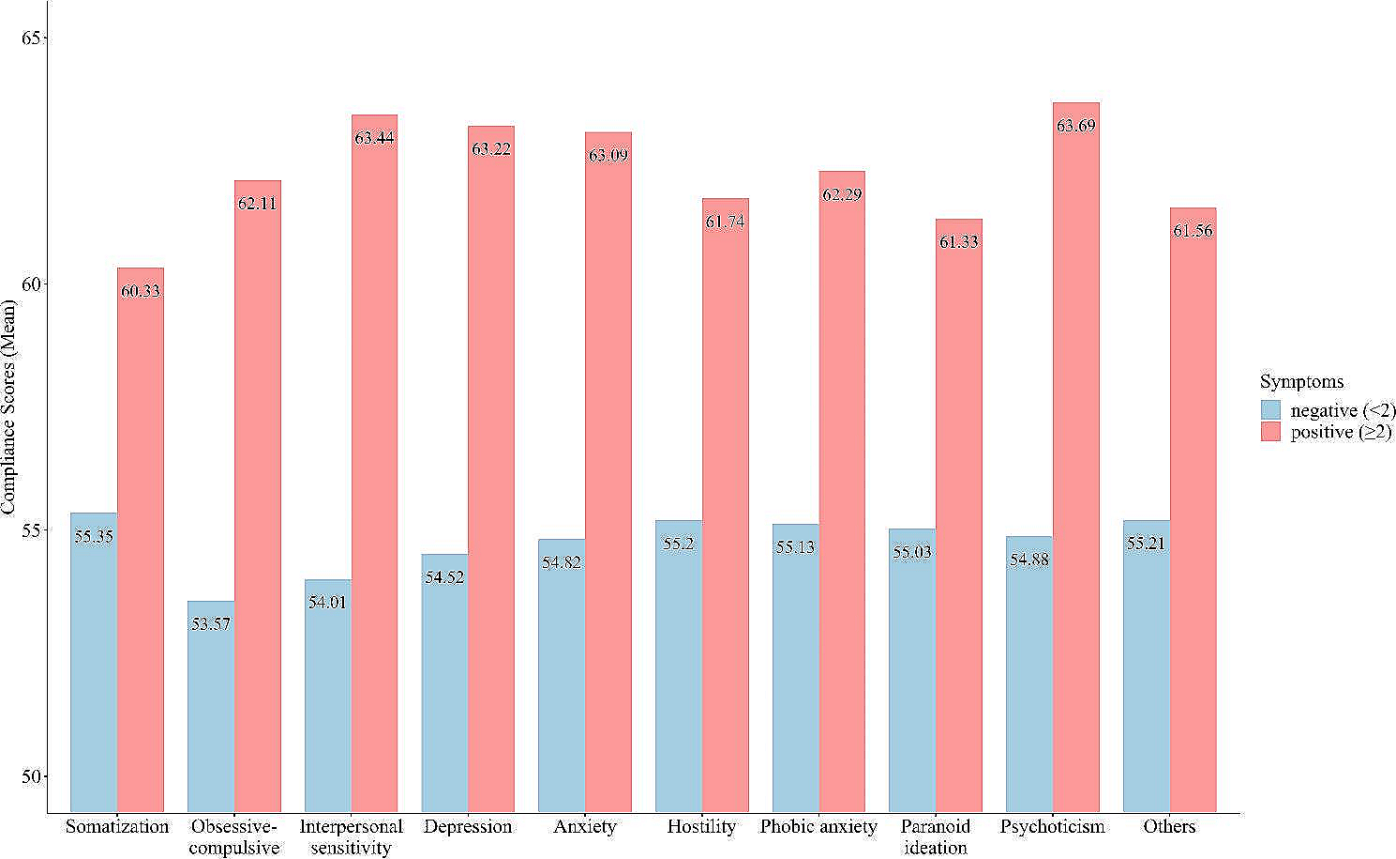
The severity of mental health problems with different degrees of trait compliance. The numbers on the histogram represent the means of the GCS total scores

For mental-health-related variables, the correlation results revealed that the GCS total scores and scores of all three factors were positively correlated with anxiety, depression, negative affect, SCL-90 total scores, and negatively correlated with self-esteem and positive affect. Among the factor scores, factor 1 showed the strongest relationship with all the variables, as revealed by its largest correlation coefficients. Furthermore, the relationships between the GCS total scores and SCL-90 factor scores were examined through descriptive means. As Fig. 2 shows, people with compliance score higher than 63 are more possible to exhibit different kinds of mental health symptoms, for example, somatization, hostility, paranoia, anxiety, depressive symptoms and so on.

**Table 5:** Correlations between estimated GCS scores and external variables (*N* = 8,632)

			GCS			
External variables	Total	Factor 1		Factor 2		Factor 3
Anxiety	**0.405** ^***^	**0.390** ^***^			0.255^***^	0.208^***^
Depression	**0.385** ^***^	**0.381** ^***^			0.227^***^	0.167^***^
Negative affect	**0.373** ^***^	**0.373** ^***^			0.169^***^	0.236^***^
SCL-90	**0.363** ^***^	**0.368** ^***^			0.188^***^	0.174^***^
Positive affect	− 0.293^***^	**− 0.309** ^***^			− 0.136^***^	− 0.197^***^
Self-esteem	− 0.284^***^	**− 0.316** ^***^			− 0.105^***^	− 0.105^***^
Extraversion	− 0.251^***^	− 0.265^***^			− 0.163^***^	− 0.105^***^
Agreeableness	− 0.097^***^	− 0.100^***^			0.023^***^	− 0.136^***^
Conscientiousness	− 0.283^***^	− 0.277^***^			− 0.162^***^	− 0.180^***^
Openness	**− 0.351** ^***^	**− 0.324** ^***^			− 0.257^***^	− 0.266^***^
Neuroticism	**0.386** ^***^	**0.381** ^***^			0.195^***^	0.254^***^

*Note.* GCS = Gudjonsson Compliance Scale. Factor 1: Difficulty in coping with authority and conflict; Factor 2: Eagerness to meet expectations; Factor 3: Social acceptance. Correlations > 0.30 are in bold font

*** *p* < .001

### Sensitivity analysis

Multiple regression analysis was carried out in three steps using total compliance as the dependent variable. Model 1 included neuroticism, extraversion, agreeableness, conscientiousness, and openness as independent variables, Model 2 added self-esteem as the independent variable, and Model 3 added generalized anxiety and depression as independent variables. Findings revealed that the Big Five traits explained 28% of the variance (adjusted R^2^) in the overall compliance score, while the self-esteem and mental health variables explained additional 3% and 4% of the variance (adjusted R^2^) in the overall compliance score, respectively. In addition, multiple regression analyses using generalized anxiety and depression as dependent variables were conducted in sequence to test the reversed association. Model 1 included the Big Five traits as independent variables, Model 2 added self-esteem as the independent variable, and Model 3 added compliance as the independent variable. For generalized anxiety, the neuroticism, extraversion, and conscientiousness explained 26% of the variance, while the self-esteem and compliance explained additional 3% and 4% of the variance in the overall anxiety score, respectively. For depression, the neuroticism, extraversion, conscientiousness and agreeableness explained 23% of the variance, while the self-esteem and compliance explained additional 7% and 2% of the variance in the overall depression score, respectively.

## Discussion

To our knowledge, this is the first study to assess the psychometric properties of the Chinese version of GCS as a measure of trait compliance. Based on the EFA results, the Chinese GCS showed a 3-factor structure with the remaining 18 items. The 3-factor model was further tested using CFA in Sample 2, which demonstrated an acceptable fit. Reliability, measurement invariance, and correlational tests were conducted on the two combined samples. Reliability was supported by the moderate-to-good internal consistency of the three-factor scales and good internal consistency of the full scale. Furthermore, tests of measurement invariance found that all three levels of measurement invariance were supported across sex, ethnicity, and data waves. Convergent validity and the positive relationships between scores of the GCS and several mental health problems were supported by the correlational analyses. These results are encouraging and support the equivalent use of the GCS with a 3-factor structure for males and females, as well as for Han Chinese people and other ethnic minorities in China.

Examination of the best-fitting underlying factor structure of the Chinese GCS revealed a 3-factor structure, which was consistent with the original version of the GCS. However, the Chinese and English versions were not identical in terms of the meaning of each factor and item placement within the factors, which may reflect the potential differences in the construct of compliance between Chinese and Western cultures. Specifically, the three factors found in the Chinese population were named “difficulty in coping with authority and conflict,” “eagerness to meet expectations,” and “social acceptance.” The meanings of the first two factors were similar to those used in previous studies [8, 13]. Difficulty in coping with authority and pressure refers to the difficulties the individual has in coping with pressure, which reflects fear and apprehension when in the company of authority figures and the tendency to avoid conflict when under pressure. Eagerness to meet expectations means that an individual prefers to do what is expected. Unlike the rather obscure factor 3 in the original version of the GCS, the third factor found in this study seems to reflect an aspect of social conformity and acceptance. Its meaning is the same as the fourth factor found in females in a previous study, which reflects an individual’s desire for social acceptance from peers or friendship groups [13]. The three reverse-scored items (items 17, 18 and 19) still loaded significantly on factor 3 in this study, while item 4 (“*I tend to give in to people who insist that they are right*”) from the original factor 3 loaded significantly onto factor 1. Considering that in Chinese culture, giving in to people who insist that they are right represents the tendency to give in under pressure, item 4 was recategorized into factor 1 (Difficulty in coping with authority and pressure). In addition, item 13 (“*When I am uncertain about things I tend to accept what people tell me*”), item 14 (“*I generally try to avoid confrontation with people*”) and item 15 (“*As a child I always did what my parents told me*”) from the original factor 1 loaded significantly onto factor 2 in this study. In Chinese culture, generally avoiding confrontation and doing what parents ask is encouraged by many people as a way of promoting harmony in relationships [43], which reflects more of the general tendency to meet others’ expectations than difficulty in coping with pressure. Thus, these items were recategorized into factor 2 (eagerness to meet expectations). Finally, item 6 (“*I try very hard not to offend people in authority*”), item 7 (“*I would describe myself as a very obedient person*”) and item 10 (“*I try to please others*”) from the original factor 2 had significant loadings on factor 1 in this study. As the meaning of pleasing others partly overlaps with the meaning of obedience to authority in Chinese culture, these items were recategorized into factor 1. In addition, item 11 (“*Disagreeing with people often takes more time than it is worth*”) and item 20 (“*When I was a child I sometimes took the blame for things I had not done*”) were omitted, as their factor loadings or communalities were not acceptable and their meanings are far from the meaning of compliance in Chinese culture.

Correlational analyses supported the positive relationships between compliance and mental health problems. The total GCS score was positively correlated with all mental health problems included in this study, some of which have been reported in previous studies [3]. Especially, the results indicated that people with compliance score higher than 63 exhibited higher psychological symptoms, for example, somatization, hostility, paranoia, anxiety, depressive symptoms and so on. All three factors correlated positively with mental health outcomes, with factor 1 (difficulty in coping with authority and pressure) having the strongest positive relationship with anxiety, depression, negative affect, and psychological distress. These findings suggest that higher levels of compliance, especially higher levels of difficulty in coping with authority and pressure, are associated with higher levels of mental health problems. Consistent with previous research [3, 44], compliance was positively correlated with anxiety. As suggested by Gudjonsson et al. (2002), people with anxiety symptoms tend to avoid conflict and confrontation. Compliant behaviors may reduce feelings of anxiety in the short term but may increase depression, negative affect, and psychological distress in the long term when people comply with things with which they may silently disagree [3]. Moreover, the total and factor scores of the GCS were negatively correlated with self-esteem and extraversion and positively correlated with neuroticism. These findings are consistent with those of previous studies [3, 24]. This study also found that mental health variables and self-esteem contributed significantly to variance in compliance beyond the effects of neuroticism and extraversion. In addition, compliance also contributed significantly to variance in mental health variables beyond the effects of Big Five traits and self-esteem. Regarding self-esteem, individuals with low self-evaluation are more likely to lack the confidence needed to resist the demands and requests imposed on them by others, especially authority figures [25]. Moreover, they may demonstrate a strong desire to please others to seek social approval, which either boosts or sustains their self-esteem [3].

Examination of measurement invariance supported the equivalent use of the GCS with a 3-factor structure for males and females in China, which was inconsistent with the study by Drake & Egan (2017). In their study, the GCS was found to measure different aspects of compliance across males and females in a sample consisted of 691 participants in UK. Specifically, the best-fitting model for males was comprised of 3 factors: eagerness to meet expectations, goal/reward-orientated obedience, and difficulty coping with pressure. On the other hand, the examination of compliance in females identified a four-factor structure consisting of fear of pressure, eagerness to please, coping with authority, and social acceptance. The structure for females was similar to the three-factor structure observed in our study, with the exception that two factors, fear of pressure and coping with authority, were combined into a single factor (factor 1) in our analysis. However, the factor structure for males was not replicated in our study, as both the meanings and item placement of the factors differed. Our findings supported the *priori* assumption that the GCS is a gender-invariant instrument [13]. It is possible that compliance manifests similarly across genders in China, given that Chinese culture places a greater emphasis on compliance for both males and females in a more consistent and absolute manner, in contrast to Western cultures [19].

This study translates and validates an important and well needed tool (GCS) measuring trait compliance for people in Chinese-speaking societies. It has enriched our understanding of compliance, especially in the context of cultural differences, and broadened the theoretical framework of compliance research. In addition, the significantly positive relationship between compliance and mental health problems may inform the future development of mental health interventions targeted at reducing unnecessary compliance. Furthermore, the validated scale could be used to identify individuals which are at risk for developing mental health symptoms (e.g., using the cutoff value of 63 as a reference for clinical psychiatric assessments).

This study has several limitations. First, the findings were based on samples of college students from two urban areas of China, which may restrict the generalizability of the results to a broader Chinese population. Future investigations should encompass more representative samples that better reflect Chinese society as a whole. Second, the cross-sectional nature of this study limits its conclusions regarding the causal inferences on the associations between compliance and mental health. Future research should explore the longitudinal associations between compliance and variables related to mental health and personality. Third, test-retest reliability was not examined in this study, this should be addressed by a test-retest design in future research. Despite these limitations, the current study has several strengths, including its robust statistical methodology and large sample size.

## Conclusion

The Chinese version of the GCS has demonstrated good psychometric properties. The results indicated that the 3-factor structure fit the data, demonstrating strong measurement invariance across sex, ethnicity, and data waves. Therefore, the Chinese version of the GCS appears to be a promising instrument for measuring trait compliance and could promote both assessment and research on compliance in the Chinese population in the future.

## Data Availability

The datasets used and/or analysed during the current study are available from the corresponding author on reasonable request.

## Declarations

For research involving human participants. All participants remained anonymous and were informed that they could withdraw from the survey at any time before submitting their responses. The participants provided informed consent before completing the survey.

## Abbreviations

GCS: Gudjonsson Compliance Scale
GAD-7: Generalized Anxiety Disorder-7
PHQ-9: Patient Health Questionnaire-9
PANAS: Positive and Negative Affect Schedule
SCL-90: Symptom Checklist 90
RSES: Rosenberg Self-Esteem Scale
EFA: Exploratory Factor Analysis
CFA: Confirmatory Factor Analysis
CFI: Comparative Fit Index
TLI: Tucker–Lewis Index
RMSEA: Root Mean Square Error of Approximation
SRMR: Standardized Root Mean Square Residual
AIC: Akaike Information Criterion
BIC: Bayesian information criterion
KMO: Kaiser Meyer-Olkin
